# Reducing Employee Injuries from Aggressive Patient Behavior at Children’s Hospital by Implementing a Behavioral Response Team

**DOI:** 10.1097/pq9.0000000000000790

**Published:** 2025-01-20

**Authors:** Julia A. Martorana, Debrea M. Griffith, Carmel Eiger, James J. Maurer, Amanda Burnside, Aron C. Janssen, Alba Pergjika, Jennifer A. Hoffmann

**Affiliations:** From the * Center for Quality and Safety, Ann & Robert H. Lurie Children’s Hospital of Chicago, Chicago, Ill.; † Ann & Robert H. Lurie Children’s Hospital of Chicago, Chicago, Ill.; ‡ Clinical and Organizational Development, Ann & Robert H. Lurie Children’s Hospital of Chicago, Chicago, Ill.; § Pritzker Department of Psychiatry and Behavioral Health, Ann & Robert H. Lurie Children’s Hospital of Chicago, Chicago, Ill.; ¶ Department of Psychiatry and Behavioral Sciences, Northwestern University Feinberg School of Medicine, Chicago, Ill.; ‖ Department of Pediatrics and Department of Medical Social Sciences, Northwestern University Feinberg School of Medicine, Chicago, Ill.; ** Division of Emergency Medicine, Department of Pediatrics, Ann & Robert H. Lurie Children’s Hospital of Chicago, Chicago, Ill.

## Abstract

**Background:**

Among hospitalized children, episodes of aggressive patient behavior place healthcare staff at risk for serious injuries. By implementing a behavioral response team at a children’s hospital, we aimed to reduce monthly employee injuries related to aggressive patient behavior from 3.4 to 2.4 per 1,000 acute care visits during 12 months.

**Methods:**

At a children’s hospital, a multidisciplinary team used quality improvement methodology to implement a behavioral response team that provided proactive and reactive support to staff caring for children at risk for aggressive behavior. Full-scale implementation occurred in July 2022. We measured days between Occupational Health and Safety Administration (OSHA)-recordable employee injuries related to aggressive patient behavior and total monthly employee injuries related to aggressive patient behavior per 1,000 acute care visits (emergency department visits and/or hospitalizations) by patients 3 years of age or older.

**Results:**

In the year after full-scale implementation, an average of 101 BRT rounds and 17 reactive responses occurred per month. The maximum number of days between OHSA-recordable employee injuries related to aggressive patient behavior increased from 163 days in the year before full-scale implementation to 271 days in the following year. Monthly employee injuries related to aggressive patient behavior decreased from 3.4 to 1.7 injuries per 1,000 acute care visits by patients 3 years of age or older.

**Conclusions:**

The BRT model, which provides proactive and reactive support to hospital staff caring for children at risk for aggressive behavior, should be considered a strategy to reduce employee injuries and promote workplace safety.

## INTRODUCTION

In the last 2 decades, pediatric emergency department (ED) visits for mental health conditions increased by 60%, and pediatric hospitalizations for mental health conditions have increased by more than 25%.^1,2^ Due to barriers accessing mental health services, children with mental health conditions are increasingly experiencing boarding (extended lengths of stay in EDs and inpatient medical units) while awaiting psychiatric admission.^3,4^ During these stays, some children display aggressive behaviors, such as hitting or biting, which place healthcare workers at risk for serious injuries.^5–7^ Compared with workers in other industries, healthcare workers are 5 times more likely to suffer workplace violence injuries.^8^

To promote workplace safety, a behavioral response team (BRT) consisting of trained personnel can provide proactive and reactive support to hospital staff caring for patients at risk for aggressive behavior.^9^ In adult hospitals, BRT models have reduced staff injuries, decreased security involvement, and reduced use of restraints and seclusion while also improving staff knowledge and self-efficacy related to behavioral escalation.^9,10^ The BRT model has recently been studied in a few children’s hospitals.^11–14^ At 1 children’s hospital, staff felt more supported in caring for patients with behavior needs,^13^ whereas another children’s hospital reported reductions in employee injuries when a BRT was implemented alongside other interventions.^15^

As presentations to our hospital for mental health concerns increased,^16^ hospital leadership identified the need to develop a centralized, standardized response to patient aggression to support patients and staff and mitigate employee harm. Here, we describe the design, implementation, and evaluation of proactive and reactive components of a hospital-wide BRT. Through quality improvement (QI) methodology, we aimed to reduce the monthly rate of employee injuries related to aggressive patient behavior by 30%, from 3.4 to 2.4 injuries per 1,000 acute care visits per month, for 12 months from July 2022 to June 2023.

## METHODS

### Context

The setting was an urban academic children’s hospital with approximately 1,500 annual mental health ED visits, 350 annual hospitalizations to medical units for boarding, and a 12-bed inpatient psychiatric unit. In the ED, psychiatric social workers are available 24 hours/d to conduct mental health evaluations, with telephonic support from child and adolescent psychiatrists and psychologists. A team of child and adolescent psychiatrists and psychologists provides consultation services for medical inpatient units.

### Interventions: BRT Design

In September 2021, we convened a BRT design team with representation from psychiatry, hospital medicine, inpatient nurses and nursing assistants, the ED, child life specialists, and security. The design process incorporated principles from the model for improvement.^17^ The team performed a current state analysis and developed a key driver diagram to delineate barriers to responding to patient aggression. Informed by this analysis, the team designed 2 BRT components: (1) BRT rounding, in which a behavioral health expert conducts daily rounds with frontline staff caring for children at risk for aggression and (2) BRT STAT, a multidisciplinary bedside response to aggressive behavior, followed by a debrief to facilitate learning and identify improvement opportunities.

We used 3 inclusion criteria to identify patients for BRT rounding: (1) a positive response to a screening question that nurses asked parents/guardians upon admission, “Does your child have a history of aggression or do you have any concerns about your child handling the stress of medical care in an unsafe or aggressive way, such as: hitting, punching, biting, throwing objects, kicking, self-injury, or other behaviors?,” (2) activation of an agitation order set that included medications for management of acute agitation and physical restraints, or (3) order placement for a patient safety attendant with an indication of “agitation/aggression.” The team worked with informatics specialists to create a visual alert and dedicated patient list in the electronic health record for patients who met the criteria for BRT Rounding. The activation criterion for a BRT STAT was “destructive, escalating patient behavior,” including imminent risk to self or others, being combative, assaulting staff/family/visitors, moving or throwing large objects, destroying property, inadequate response to attempted de-escalation, and/or staff feeling that they need additional support.

**Fig. 1.**
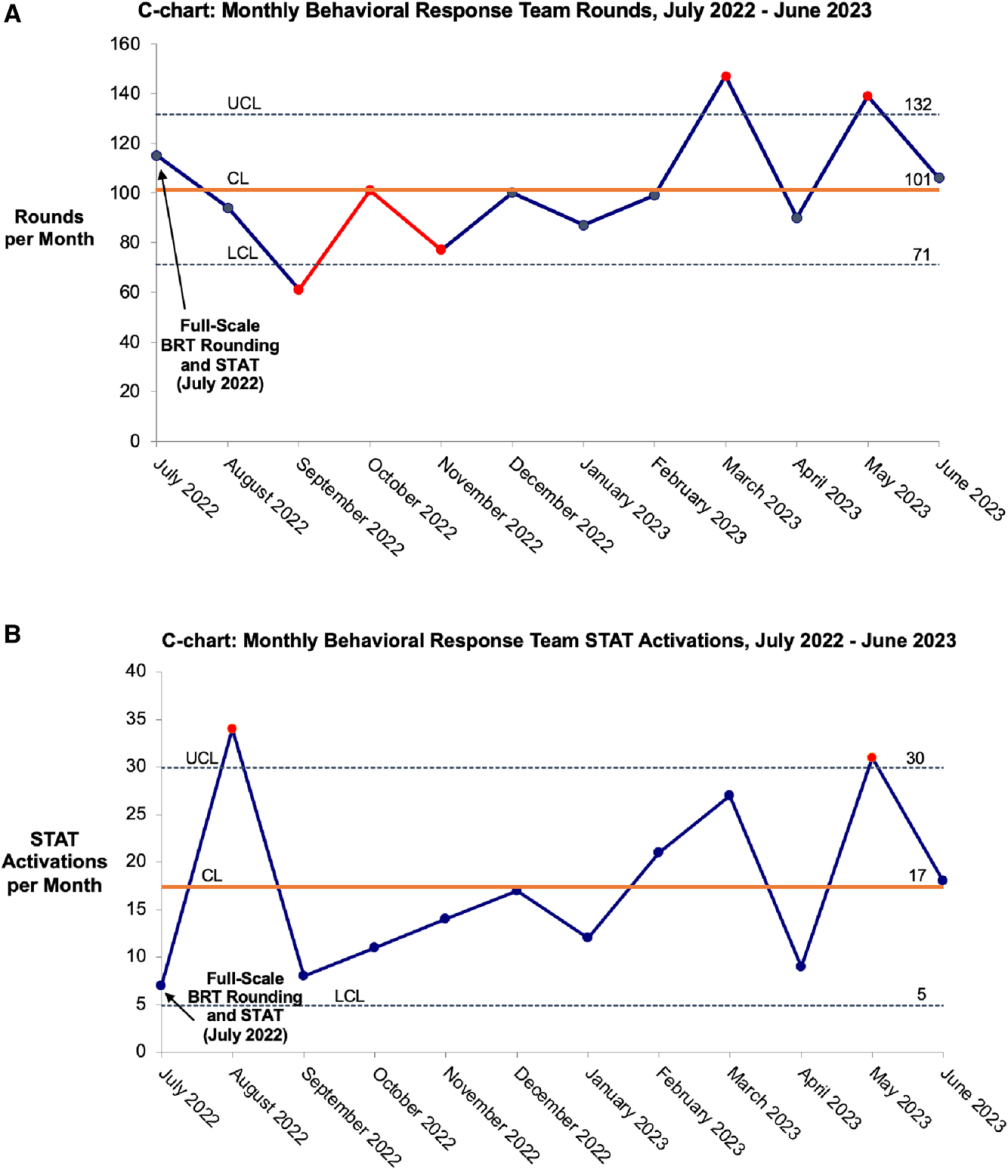
C-charts: monthly BRT rounds and STAT activations, July 2022–June 2023. A, BRT rounds were conducted an average of 101 times per month (range: 61–147). Criteria for special cause variation were met from September to November 2022 with 2 of 3 points outside the 2-sigma control limit and in March and May 2023 due to individual points above the upper control limit. B, A BRT STAT response was activated an average of 17 times monthly. Criteria for special cause variation were met in August 2022 and May 2023 due to individual points above the upper control limit. CL, Control limit; LCL, Lower control limit; UCL, Upper control limit.

**Fig. 2.**
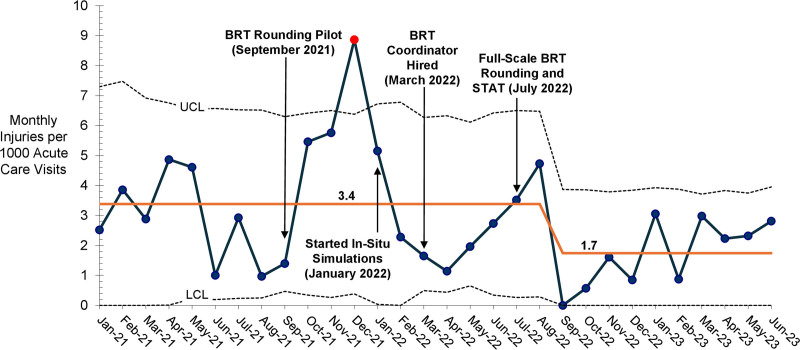
U-chart: Monthly employee injuries related to aggressive patient behavior per 1,000 acute care visits, January 2021–June 2023. Each point reflects monthly employee injuries of any severity related to aggressive patient behavior per 1,000 acute care visits (ED visits and/or hospitalizations on medical inpatient units) by patients 3 years of age or older. The orange line represents the average monthly employee injuries per 1,000 acute care visits by patients 3 years of age or older. CL, Control limit; LCL, Lower control limit; UCL, Upper control limit.

The team developed a BRT implementation toolkit which included a BRT Rounding Communication Tool that listed rounding inclusion criteria and a rounding script (Table 1), BRT STAT roles and responsibilities (Table 2), a BRT STAT script (Table 3), a standardized debrief form (**see Figure 1, Supplemental Digital Content 1**, which displays the debrief form. http://links.lww.com/PQ9/A634), and a BRT workflow algorithm (**see Figure 2, Supplemental Digital Content 1**, which displays the algorithm. http://links.lww.com/PQ9/A634).

**Table 1.** BRT Rounding Communication Tool

Rounding inclusion criteria	• Positive answer to the following Behavioral Support Guidelines* screening question asked by the nurse to the parent/guardian upon admission: • “Does your child have a history of aggression, or do you have concerns about your child handling the stress of medical care in an unsafe or aggressive way?” • Patient with launched ED agitation algorithm, triggered by use of ED agitation order set • Patient with launched acute care agitation algorithm, triggered by use of acute care agitation order set • Patient safety associate order with an indication of “agitation/aggression”
Initiation of rounding	• Behavioral Response Team Lead to use contact information on patient door to contact nurse and certified nursing assistant • Introduce service if not familiar with team: “I am from the Behavioral Response Team. We are conducting proactive rounding to help support staff in caring for patients with challenging behaviors. I just have a few quick questions for you if you have time.”
Rounding script for behavioral response team lead	1. How are you doing? How has the patient been doing today? 2. Review the patient’s Behavioral Support Guidelines* • Has anything made the patient agitated or anxious today? • Has the patient been aggressive today? Any new concerns or aggressive episodes since yesterday? • If yes: What happened? What seemed to help de-escalate? How do we think the episode could be prevented in the future? Provide de-escalation techniques sheet to staff, if needed. • If no: What seems to be helping keep patient calm? How have they been spending the day? What has gone well? • Were there any episodes of restraint or seclusion? • Have any injuries (to patient or staff) occurred? Have they been reported? If not, encourage reporting via Safety Event Reporting System and/or Employee Incident Report. 3. Are you familiar with the PRN medication plan for this patient? Do you have availability of PRN medication for management of aggressive behavior if needed? • Have you administered any PRN medications? How frequently are you having to use them? What was the response? • What behaviors does this patient display before escalation/need of PRN medication? Remind bedside nurse/certified nursing assistant that PRNs should be given as the patient is becoming agitated/aggressive, not after. 4. Do we need additional consults ordered? (ie occupational therapy, physical therapy, speech, child life, music therapy, art therapy, education) • If a consult is needed, team lead to connect with medical resident or psychiatry consult-liaison trainee after rounds to place consult order. 5. Are patient safety associate needs met for this patient? Have the patient safety associates been inside or outside of the room? Has that been consistent across shifts? • If a patient safety associate is needed, ask bedside nurse to enter the patient safety associate order with appropriate indication (ie, “agitation/aggression”). 6. Discuss elopement risk and strategies to mitigate: • No shoes, wearing scrubs, belongings secure, and patient does not have access to them • No personal electronics • Security aware patient is an elopement risk (if needed) • Security outside door (if needed) 7. How have you partnered with the family in care of this patient? • Review visitation guidelines for this patient • Have caregivers been present today? Has that seemed to be helpful or not for the patient’s behavior? 8. Are there any outstanding questions that you need answered to be able to safely do your job today? How else can I be helpful or support you? • Review protective gear available and where to find it. 9. Who on your unit can you reach out before the next rounds if additional questions arise? You may also reach me at the pager number in my note (team lead contact info), the patient’s primary psychiatry provider, or the psychiatry provider on call. 10. If family member is present at bedside, review and update questions in the Behavioral Support Guidelines* with them to learn about the patient’s triggers and previously helpful de-escalation strategies.

* Behavioral Support Guidelines are individualized care plans in the electronic health record that incorporate patient-specific triggers and de-escalation techniques. A positive answer to the screening question prompts completion of an individualized care plan.

PRN, Pro re nata.

**Table 2.** BRT STAT Roles and Responsibilities

Role	Responsibilities
BRT lead	• Lead team through BRT STAT script • Review patient Behavioral Support Guideline* and facilitate removal of triggers • Schedules, conducts, and documents debrief
Bedside nurse	• Ensure medical resident or attending and charge nurse are aware of BRT STAT • Describe Situation, Background, Assessment, and Recommendation when team arrives • Assessment, monitoring, and documentation of restraints • Administer any medications ordered • Observe patient airway, breathing, circulation • Ensure interpreter if needed • Ensure nursing leadership is aware of behavioral health crisis • Report any injuries that occurred
Patient safety associate (“sitter”)	• Call bedside nurse to alert them of BRT STAT • Describe additional context/events leading up to current state of escalation (2–3 sentences if needed after nurse)
Medical provider (medical resident, advanced nurse practitioner, or attending)	• Call medical attending to alert them of BRT STAT • Share contraindications to agitation medications or restraints • Order STAT medications if needed • Order mechanical restraints within 10 min of placement, if needed • Complete mechanical restraint documentation, if needed • Discuss differential for etiology of behavioral event based on: direct clinical assessment, details about medical/psychiatric history and hospital course, pertinent test results • Recommend obtaining vitals, laboratory results, or studies as indicated
Security	• Available for additional physical support • Clear area near patient room of observers
Psychiatry fellow (In person until 5 pm; STAT page after hours)	• Provide recommendations for medications • Evaluate need for mechanical restraints
Floor charge nurse	• Situational awareness for rest of unit (ie, close other patient doors near escalating patient room) • Ensure that the nurse’s other patients have adequate care coverage • Page team members if they have not arrived within 10 min • Move personal protective equipment cart to outside of patient’s room
Chaplain	• Available as a staff resource during the debrief process

* Behavioral Support Guidelines are individualized care plans in the electronic health record that incorporate patient-specific triggers and de-escalation techniques.

**Table 3.** BRT STAT Script

Response Stage	Script and Specific Steps
Arrival	• Team lead: “Is anyone hurt?” (patient, family, staff). Attend to needs as appropriate. • Team lead: “The purpose of this huddle is to address behavioral concerns and come up with a mitigation plan.” • Roll call: Name and title. If members missing, call or page according to roles and responsibilities. • Bedside nurse: Situation, Background, Assessment, Recommendation about current patient status • Patient safety associate: Additional context of events leading up to BRT activation if needed (2–3 sentences)
Mitigation	• Decide who should be in the room versus outside of the room and if closing the door would be appropriate/safe • Assess environment and remove triggers/risks • Use de-escalation techniques (see agitation algorithm, psychiatry note, Behavioral Support Guidelines,* multidisciplinary note) • Review any contraindications to medications or restraints and discuss differential for etiology of behavioral event • Review PRN medication plan and need for medication use (see agitation algorithm or personalized agitation plan) • Discuss need for mechanical restraints (see restraints guidance in agitation algorithm)
Next steps	• Team lead summarizes plan and team members confirm actions/comfort with plan • Complete team debrief (immediately, if possible, otherwise schedule for same shift) • Update Behavioral Support Guidelines* and PRN medication plan, if needed • Bedside nurse submits safety event report (if patient/staff injury occurred) • Team lead completes BRT flowsheet documentation

* Behavioral Support Guidelines are individualized care plans in the electronic health record that incorporate patient-specific triggers and de-escalation techniques.

PRN, pro re nata.

Next, BRT components were piloted and iteratively refined. In September 2021, BRT rounding was piloted with a single child psychologist and modified via plan-do-study-act cycles based on feedback from staff and families. In January 2022, the team initiated a failure mode and effects analysis^18^ and used simulation to identify vulnerabilities in the BRT STAT process and generate solutions for prioritized failure modes. We conducted 20 in situ simulations on day and night shifts in the ED and inpatient units from January to June 2022, with 142 participants. Simulations introduced BRT processes to clinical staff, detected process vulnerabilities, and tested proposed solutions. Simulations focused on when and how to activate a BRT STAT and how each team member should respond. During simulations, an observer recorded timestamps (eg, time of activation call, time that each responder arrived at bedside) and noted improvement opportunities. Simulation debriefs were conducted using the Promoting Excellence and Reflective Learning in Simulation tool, which incorporates scripted language to promote self-assessment, focused discussion, and directive feedback.^19^ Action items that emerged from the failure mode and effects analysis included disseminating an online BRT learning module (completed by 2984 staff), adding psychiatric medications to medication dispensing machines throughout the hospital, revising an environmental safety checklist, placing behavioral personal protective equipment carts throughout the hospital, and creating an electronic health record flowsheet for BRT documentation.

In March 2022, a full-time BRT program coordinator was hired to train and manage 12 BRT Leads (registered nurses and milieu therapists), enabling 24-hour staff coverage. Leads rotated between shifts dedicated to BRT responsibilities and clinical duties within the inpatient psychiatric unit. In July 2022, full-scale implementation of BRT Rounding and BRT STAT processes occurred in the ED and medical inpatient units. We integrated simulations to teach BRT processes into de-escalation training offered to all clinical staff, with 38 classes completed from February 2022 to June 2023 involving 387 participants.

The BRT program coordinator completed quarterly case reviews and appointed select BRT design team members to review BRT Rounding and BRT STAT data and OSHA-recordable injury events. Identified opportunities for process improvement were implemented using plan-do-study-act cycles. Changes made based on case reviews included incorporating certified child life specialists into BRT rounding and expanding BRT STAT availability to procedural and sedation services.

### Study of the Intervention

We tracked employee injuries related to aggressive patient behavior for 18 months before (January 2021–June 2022) and 12 months after (July 2022–June 2023) full-scale BRT implementation. In August 2021, we surveyed nurses to assess how supported they felt in caring for patients with complex behavioral health needs. Approximately 1 year after full-scale implementation, the same question was embedded within a follow-up survey, along with 2 new questions: (1) “How supported do you feel by the BRT?” and (2) “How valuable has BRT been to your unit?”

### Measures

The primary outcome measure was the monthly rate of reported physical employee injuries (regardless of the level of harm) related to aggressive patient behavior per 1,000 acute care visits (ED visits ± hospitalizations) by patients 3 years of age or older.^20^ Physical injuries were reported to an occupational health vendor via a standardized report form. A secondary outcome measure was the number of days between OSHA-recordable employee injuries related to aggressive patient behavior in the ED and inpatient medical units. Injuries are considered OSHA-recordable if they result in medical treatment beyond first aid, days away from work, restricted work, transfer to another job, or loss of consciousness or death.^21^ Occupational health staff reviewed reported OSHA-recordable events to ensure consistency with this definition.

Process measures included (1) number of BRT rounds and unique patients receiving BRT rounding per month, (2) monthly BRT STAT activations, (3) the percentage of BRT STAT calls among patients already identified as high risk for behavioral escalation via BRT Rounding criteria, (4) the percentage of BRT STAT calls with the entire team at bedside, and (5) the percentage of BRT STAT activations with a debrief performed. As a balancing measure reflecting resource utilization (as a proxy for program costs), we considered time spent by BRT Leads in responding to BRT STAT activations. Specifically, we measured the time from BRT STAT activation to the arrival of the team lead to the patient room, time spent in de-escalation, and time spent debriefing. As a second balancing measure, we monitored physical restraint application during acute care visits for patients who met the inclusion criteria for BRT Rounding.

### Analysis

The monthly rate of all reported aggressive patient behavior employee injuries per 1,000 acute care visits by patients 3 years of age or older was displayed and monitored on a U-chart. We included data points between the BRT Rounding pilot and full-scale BRT implementation in baseline centerline calculations. Days between OHSA-recordable injuries were observed on a T-chart for rare events. BRT rounding and STAT activations per month were monitored on C-charts. We defined special cause variation according to standard rules^,22,23^ which included individual points outside control limits and 8 consecutive points above or below the centerline. Statistical process control charts were generated with QI Charts (Process Improvement Products, San Antonio, TX, version 2.0.23) and Microsoft Excel 2021 (version 2302).

We summarized baseline and follow-up survey responses with descriptive statistics. A chi-square test, with a significance level of *P* <0.05, was used to compare the proportion of nurses who felt somewhat or very supported in addressing patients with complex behavioral health needs at baseline versus follow-up.

### Ethical Considerations

This study was reviewed by the hospital’s institutional review board and deemed QI, not human subjects research.

## RESULTS

During the 12 months after full-scale BRT implementation (July 2022–June 2023), BRT Rounds were conducted an average of 101 times per month (range: 61–147) with an average of 28 unique patients per month (range: 20–42). The BRT STAT response was activated 209 times, averaging 17 responses per month. Trends in monthly counts of BRT Rounds generally mirrored trends in counts of BRT STAT activations; for example, May 2023 exceeded the upper control limit for both rounds and STATs (Fig. 1). Of BRT STAT responses, 187 (89.5%) were called on patients who met the inclusion criteria for BRT rounding. The full team was documented as present at the bedside for all BRT STAT responses. A debrief was performed for all but one BRT STAT response. On average, the BRT team lead arrived in 4 minutes, 41 minutes were spent in de-escalation, and debriefs lasted 10 minutes. The frequency of physical restraint use remained within control limits throughout the study period.

After the BRT Rounding pilot, monthly employee injuries of any severity related to aggressive patient behavior in the ED and medical inpatient units remained above the centerline for 4 months, including one point in December 2021 that exceeded the upper control limit. After full-scale BRT implementation, employee injuries were above the centerline for 1 month in August 2022, followed by a decrease resulting in a centerline shift from 3.4 to 1.7 employee injuries per 1,000 acute care visits by patients 3 years of age or older (Fig. 2).

**Fig. 3.**
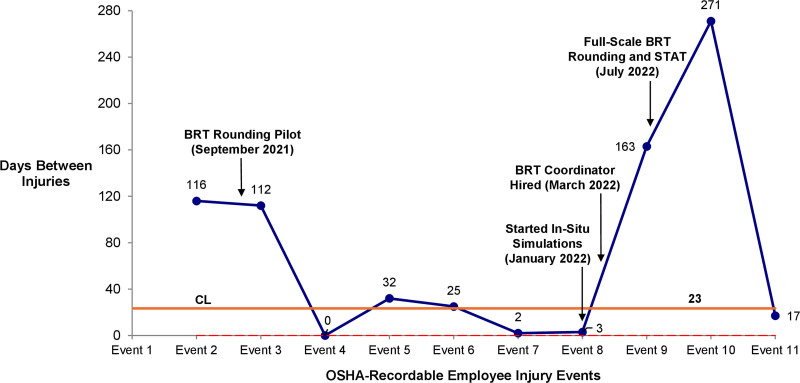
T-chart: days between OSHA-recordable employee injuries related to aggressive patient behavior, January 2021–June 2023. In this rare event chart, each point represents 1 OSHA-recordable employee injury related to aggressive patient behavior in the ED or a medical inpatient unit. The *y* axis represents the days since the last OSHA-recordable injury, and the orange line represents the average days between injury events.

In the ED and medical inpatient units, there were 9 OSHA-recordable employee injuries in the year before full-scale BRT implementation and 2 OSHA-recordable employee injuries in the year following full-scale implementation. Of the 2 OSHA-recordable injuries that occurred after full-scale implementation, one resulted from a patient with no history of aggression who did not meet the criteria for BRT Rounding. The second happened during a BRT STAT response and involved a patient who had received support from BRT rounding throughout their admission. Overall, the maximum number of days between OHSA-recordable employee injuries related to aggressive patient behavior increased from 163 days in the year before full-scale implementation to 271 days in the year following full-scale implementation (Fig. 3).

The baseline survey was completed by 50 nurses (response rate 4.2%), and the follow-up survey was completed by 193 nurses (response rate 16.1%). The percentage of nurses who reported feeling somewhat or very supported in addressing patients with complex behavioral health needs increased from 36.0% (18 of 50) to 59.9% (115 of 192) (*P* < 0.01). In the follow-up survey, among nonmissing responses, 165 (95.4%) of 173 nurses felt somewhat or very supported by the BRT, and 169 (92.9%) of 182 nurses felt the BRT was somewhat or very valuable to their unit.

## DISCUSSION

Through a rigorous process grounded in QI methodology, we developed, implemented, and evaluated a hospital-wide BRT program to provide proactive and reactive support to staff caring for children at risk for aggressive behavior. QI methodology facilitated the design of the BRT as a reliable system that led to a maximum time interval of nearly 9 months between serious employee injuries related to aggressive patient behavior after full-scale BRT implementation. Moreover, the monthly rate of employee injuries of any severity related to aggressive patient behavior decreased by approximately half following full-scale BRT implementation.

Overall, BRT Rounding criteria accurately identified most patients who later developed significant behavioral escalation. Of BRT STAT activations, only about 1 in 10 did not meet the criteria for BRT Rounding and provision of proactive support. In general, months with more rounding had more STATs, which may have reflected higher patient volume or acuity. Although limited by low response rates, survey data indicated that nurses felt significantly more supported in caring for patients with complex behavioral health needs after BRT implementation, with most nurses endorsing that the BRT was valuable to their unit. Daily BRT Rounding likely contributed to increased feelings of support.

Prior literature has suggested that underreporting employee injuries is common in hospital settings.^24,25^ For this reason, BRT Leads emphasized the importance of reporting during rounds and debriefs. Following the BRT Rounding pilot, there were 4 points above the centerline for employee injuries of any severity level, which we hypothesize were related to increased reporting, thus reflecting a more accurate baseline for events in our organization. Following full-scale BRT implementation, reported injury rates decreased with special cause variation and a centerline shift, which we suspect was related to a true decrease in injuries, given the continued emphasis on reporting.

To our knowledge, QI methodology has previously been used to evaluate BRT models at 2 children’s hospitals.^13,15^ At 1 hospital, similar to our effort, BRT STAT responses were supported by well-defined roles, a script, and a debrief.^13^ Instead of daily rounding, proactive support was offered in the form of “huddles” that required team members to recognize risk for escalation and call the hospital operator, with responders arriving within 15 minutes. The proactive and reactive support balance was 55% huddles and 45% STAT responses. Hospital staff perceptions of support and confidence in safely caring for patients with behavior needs improved, but OSHA-recordable events did not decrease.^13^ In contrast, our BRT team did not rely on having staff remember to request proactive support because electronic lists of patients who met BRT Rounding criteria were automatically generated. Moreover, our efforts averaged 101 rounds and 17 monthly STAT responses, corresponding to 86% proactive and 14% reactive responses. BRT Rounding provided just-in-time training and increased organizational capacity for frontline staff to care for patients with complex behavioral health needs, resulting in nearly 9 months without an OSHA-recordable event and a significant decrease in employee injuries of any severity.

In the second prior study of BRT implementation at a children’s hospital, a BRT was introduced with four additional interventions: aggression mitigation tools, clinical resources, advanced training in de-escalation techniques, and screening for agitation risk, coupled with standardized behavior plans.^15,26^ This approach resulted in a nearly 60% reduction in the rate of employee injuries related to aggressive patient behavior.^15^ This suggests that a BRT may function optimally when aligned with other comprehensive efforts to mitigate patient aggression. Our efforts similarly integrated behavioral carts, access to specialized BRT staff, and screening for agitation risk, resulting in reduced employee injuries. Our extensive use of simulation training was one aspect that differentiates our work. Although prior simulation efforts have focused on teaching de-escalation techniques^27^ or training BRT leads to respond,^28^ of our simulations included frontline clinical staff on day and night shifts. They guided activating a BRT STAT and enacting a coordinated team response.

Our study is subject to several limitations. As described earlier, our measures may be limited by injury underreporting. However, a strong culture of safety reporting was demonstrated by reporting aggressive patient behavior injuries, regardless of the level of harm. We focused this work on employee injuries and did not measure patient or family injuries. The survey focused on perceptions of the BRT model among nurses rather than other clinical staff, and the low response rate may have introduced nonresponse bias. Finally, although we evaluated staff time allocated to BRT STAT responses, we did not comprehensively assess the financial cost of the BRT program.

The next steps will include a greater focus on sustainment. Verbal violence and emotional harm may be valuable additions to the BRT metrics plan. Interviews with staff, patients, and families may assist in understanding the remaining opportunities for process improvement. Studies are needed to investigate whether BRT models influence staff burnout and retention rates, whether BRT models reduce disparities in pharmacologic and physical restraint use by race and ethnicity,^29–31^ and how well BRT models work for specific populations, such as children with autism spectrum disorder.^32^

In conclusion, implementing a BRT model that provides proactive and reactive support to hospital staff caring for children at risk for aggressive behavior resulted in nearly 9 months without an OSHA-recordable employee injury related to aggressive patient behavior. The rate of monthly employee injuries of any severity related to aggressive patient behavior decreased by approximately half. Thus, children’s hospitals should consider implementing a BRT model to promote workplace safety.

## ACKNOWLEDGMENTS

The authors would like to acknowledge Rebecca J. Stephen, MD, MS, and Lynn Liu, PhD, for assistance with data visualization, including development of statistical process control charts, Teresa Garcia, BS, and Victoria R. DeNardo, BSN, RN, NI-BC, for assistance with implementing electronic health record modifications, and Cynthia E. Barnard, PhD, MBA, MSJS, for review of the methodologic approach.

Supported by the National Institute of Mental Health of the National Institutes of Health under Award Number K23MH135206 [to Dr. Hoffmann]. The content is solely the responsibility of the authors and does not necessarily represent the official views of the National Institutes of Health.

Presentation at the Children’s Hospitals Solutions for Patient Safety: Spring National Learning Session, May 25–26, 2022, Denver, CO; the American Academy of Pediatrics National Conference and Exhibition, October 8, 2022, Anaheim, CA; the Children’s Hospital Association Behavioral Health Summit, October 11, 2022, Minneapolis, MN; the Institute for Healthcare Improvement Patient Safety Congress, April 11, 2022, Dallas, TX; and the 23rd International Meeting on Simulation in Healthcare, January 20–25, 2023, Orlando, FL.

## Supplementary Material


