# Supplementary material for: Reducing Employee Injuries from Aggressive Patient Behavior at Children’s Hospital by Implementing a Behavioral Response Team
Source: Pediatr Qual Saf. 2025 Jan 20;10(1):e790. doi: 10.1097/pq9.0000000000000790 (PMC11745859; doi:10.1097/pq9.0000000000000790)

Supplemental Material

Supplemental Figure 1. Standardized Debrief Form

Supplemental Figure 2. Algorithm Summarizing Behavioral Response Team Workflow

Supplemental Figure 1. Standardized Debrief Form

BRT Debrief Form

BRT Lead Name:	STAT Pager Time:
Date:	BRT Arrival Time:
Location:	Debrief Start Time:
Patient MRN:	Debrief End Time:

BRT Team Lead:

1. Were there any injuries (specify patient, staff, or family member)? YES / NO
   Remind injured person(s) to complete a SERS.

2. Did the patient meet criteria for a BRT STAT activation? YES / NO

3. What led up to the BRT STAT activation?

4. Can we identify a pattern for behavior/STAT call (e.g., triggers, time of day, where it's occurring...)? YES / NO

5. What strategies worked to de-escalate the patient? Please ensure these strategies are listed in the patient's Getting to Know Me Tips and Behavioral Support Guidelines.

6. Please indicate if any changes below could be beneficial to the patient's treatment plan.

   - PRNs:
   - PT/OT:
   - Child Life:
   - School:
   - Music:
   - Art:
   - Other:
   - Daily schedule:
   - Tip sheet at bedside:
   - Environmental changes:
   - Security outside room:
   - Review restrictions/privileges:
   - Behavioral PPE Cart:

Next Steps:

- ☐ Who is doing what follow-up? Review action item owners for each identified outstanding action
- ☐ Review employee wellness resources (e.g., Chaplain, Employee Assistance Program, RISE, wellness online portal, etc.)
- ☐ Bedside RN to complete safety event report and restraint charting if applicable
- ☐ BRT Lead to complete BRT STAT note and BRT Flowsheet documentation
- ☐ BRT Lead provided the opportunity to bedside team in identifying restraint documentation/renewal questions

Signature of Debrief Participants:

Name	Role/Job Title	Signature

Thank you!

Supplemental Figure 2. Algorithm Summarizing Behavioral Response Team Workflow

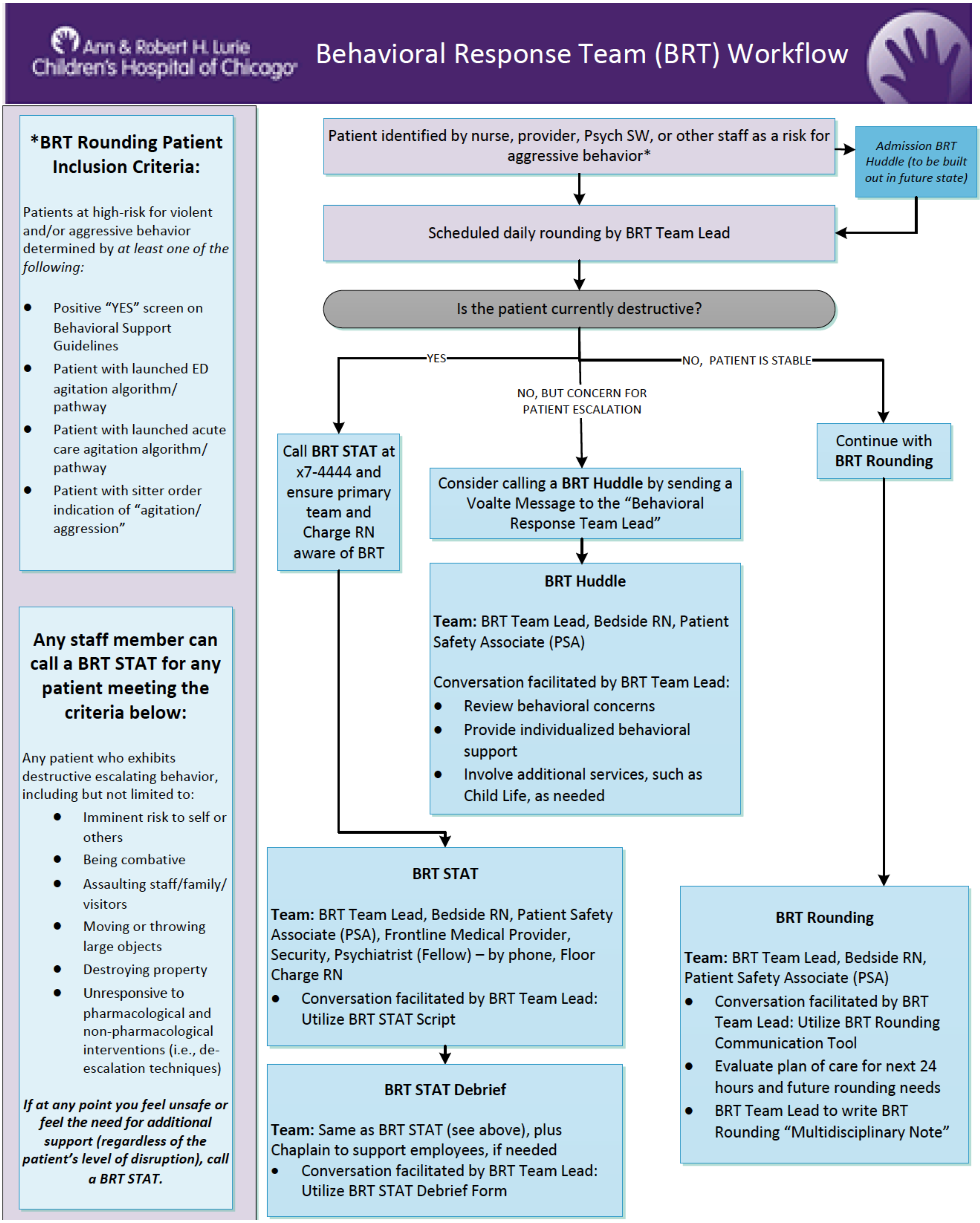

Supplement: Supplementary file 1 [file pqs-10-e790-s001.pdf]
